# Magnetic resonance imaging at 3.0-T in postmenopausal osteoporosis: a prospective study and review of the literature

**DOI:** 10.1590/0100-3984.2021.0124

**Published:** 2022

**Authors:** Mirko Trentadue, Carlo Sozzi, Luca Idolazzi, Gianluigi Lazzarini, Riccardo Sante Murano, Davide Gatti, Maurizio Rossini, Enrico Piovan

**Affiliations:** 1 Radiology Unit, Azienda ULSS 9 Scaligera, Hospital M. Magalini, Villafranca di Verona, Italy.; 2 SC Neuroradiology, ASST Carlo Poma, Mantova, Italy.; 3 Rheumatology Unit, Department of Medicine, University of Verona, Verona, Italy.; 4 Independent Researcher, self-employed Occupational Medicine specialist, Peschiera del Garda, Italy.

**Keywords:** Osteoporosis, Postmenopause, Vertebral body/pathology, Multiparametric magnetic resonance imaging/methods, Bone marrow/pathology, Osteoporose, Pós-menopausa, Corpo vertebral/patologia, Imageamento por ressonância magnética multiparamétrica/métodos, Medula óssea/patologia

## Abstract

**Objective:**

To promote advanced research using magnetic resonance imaging (MRI) in the diagnosis of and screening for osteoporosis by looking for correlations among the T-scores measured by dual-energy X-ray absorptiometry (DEXA), the apparent diffusion coefficient (ADC) values on diffusion-weighted imaging (DWI), and the T1-weighted signal intensity values.

**Materials and Methods:**

This was a prospective study of postmenopausal women with no contraindications to MRI and no history of cancer who underwent DEXA within 30 days before or after the MRI examination. A 3.0-T scanner was used in order to acquire sagittal sequences targeting the lumbar spine.

**Results:**

Thirteen women underwent DEXA and MRI. In two cases, the MRI was discontinued early. Therefore, the final sample comprised 11 patients. The ADC values and T1-weighted signal intensity were found to be higher in patients with osteoporosis. However, among the patients > 60 years of age with osteoporosis, ADC values were lower and T1-weighted signal intensity was even higher.

**Conclusion:**

It is unlikely that MRI will soon replace DEXA for the diagnostic workup of osteoporosis. Although DWI and ADC mapping are useful for understanding the pathophysiology of osteoporosis, we believe that T1-weighted sequences are more sensitive than is DWI as a means of performing a qualitative analysis of vertebral alterations.

## Abstract (pt)

**Objetivo:**

Promover pesquisas avançadas usando ressonância magnética (RM) no diagnóstico e rastreamento de osteoporose, procurando correlações entre os escores T medidos por absorciometria de raios-X de dupla energia (DEXA), valores de coeficiente de difusão aparente (ADC) na difusão e valores de intensidade de sinal ponderado em T1.

**Materiais e Métodos:**

Estudo prospectivo de mulheres na pós-menopausa sem contraindicações para RM e sem histórico de câncer que foram submetidas a DEXA 30 dias antes ou após o exame de RM. Um *scanner* 3.0-T foi utilizado para adquirir sequências sagitais direcionadas à coluna lombar.

**Resultados:**

Treze mulheres foram submetidas a DEXA e RM. Em dois casos, a RM foi interrompida precocemente. Portanto, a amostra final foi composta por 11 pacientes. Os valores de ADC e intensidade de sinal ponderado em T1 foram mais elevados nas pacientes com osteoporose. No entanto, no subgrupo de pacientes > 60 anos de idade com osteoporose, os valores de ADC foram menores e a intensidade do sinal ponderado em T1 foi ainda maior.

**Conclusão:**

É improvável que a RM substitua DEXA para a investigação diagnóstica da osteoporose no futuro próximo. Embora a difusão e o mapeamento ADC sejam úteis para a compreensão da fisiopatologia da osteoporose, acreditamos que as sequências ponderadas em T1 são mais sensíveis do que a difusão como meio de realizar uma análise qualitativa das alterações vertebrais.

## INTRODUCTION

The instrumental evaluation of osteoporosis is currently based almost exclusively on the measurement of bone mineral density (BMD), which is typically performed with dual-energy X-ray absorptiometry (DEXA). The population most affected by osteoporosis is postmenopausal women. We hypothesized that the greater intravertebral representation of adipose tissue would lead to restricted diffusion on diffusion-weighted imaging (DWI), with a subsequent decrease in the apparent diffusion coefficient (ADC) values and an increase in T1-weighted signal intensity on magnetic resonance imaging (MRI). Therefore, the reduction in ADC values and the increase in T1-weighted signal intensity would become progressively more pronounced as the BMD and T-score values on DEXA decrease.

The aim of this study was to test the aforementioned hypothesis by using high-field (3.0-T) MRI. We focused our attention on the lumbar spine, in order to determine whether MRI can be a valuable complement to bone densitometry for the early identification of individuals with osteoporosis, who are intrinsically at higher risk for vertebral fracture.

## MATERIALS AND METHODS

### Study design and population

This was a prospective study in which volunteers were recruited from among postmenopausal women who underwent DEXA and MRI for the investigation of suspected osteoporosis. Women with absolute contraindications to MRI were excluded, as were those with a history of bone tumors or bone metastases, those who underwent DEXA more than 30 days before or after the MRI examination, those with acute/subacute vertebral fractures, those with a recent history of trauma affecting the lumbar spine, and those having previously undergone surgery involving the lumbar spine. The study was approved by the local institutional review board. All participating patients gave written informed consent.

### Imaging protocol

All MRI examinations were performed in a 3.0-T scanner (Achieva; Philips Healthcare, Best, the Netherlands), with a dedicated 12-channel body coil (Sense; Philips Healthcare). The acquisition of images of the lumbar spine in three planes was followed by the positioning of a reference for the optimization of the magnetic field in the study field of view.

Each examination lasted approximately 15 min and included the spine segment between vertebra T12 and vertebra S3. The acquisition of sagittal sequences-T1-weighted turbo spin-echo (TSE); T2-weighted TSE; short-tau inversion recovery; and DWI with four b-values (b0, b300, b500, and b800)-was followed by the calculation of the corresponding ADC maps (Table 1). No contrast medium was administered. The examinations were performed and viewed by reader 1, a radiologist with 5 years of experience, and were reviewed by reader 2, a neuroradiologist with 30 years of experience, to detect any incidental finding of clinical relevance not already known to the subject.

**Table 1:** Sequence design for the study protocol.^*^

Sequence	Parameters
T1-weighted TSE	TR/TE, 555/8 ms; FOV, 160 × 268 × 49; voxel size, 1 × 1 mm; slices, 15; slice thickness, 3 mm; reconstruction matrix, 560 × 560; acquisition time, 4 min 31 s
Short-tau inversion recovery	TR/TI, 5,406/210 ms; TE, 60 ms; FOV, 160 × 268 × 49; voxel size, 1 × 1.46 mm; slices, 15; slice thickness, 3 mm; reconstruction matrix, 560 × 560; acquisition time, 4 min 30 s; saturation band positioned anterior to the spine
T2-weighted TSE	TR/TE, 4,496/110 ms; FOV, 160 × 268 × 49; voxel size, 1 × 1.25 mm; slices, 15; slice thickness, 3 mm; reconstruction matrix, 560 × 560; flip angle, 90°; acquisition time, 2 min 50 s
DWI	Four b-values (b0; b300; b500; b800); TR/TE, 2,444/56 ms; FOV, 280 × 239 × 89; voxel size, 2.05 × 2.55 mm; slices, 15; slice thickness, 5 mm; reconstruction matrix, 560 × 560; EPI factor, 57; single-shot spin-echo; acquisition time, 3 min 17 s

* All sequences obtained in the sagittal plane and in a 3.0-T MRI scanner.

TR, repetition time; TE, echo time; FOV, field of view; TI, inversion time; EPI, echo-planar imaging.

An automated analysis system (IntelliSpace Portal; Philips Healthcare) and picture archiving and communication system software (Carestream Vue PACS; Carestream Health, Rochester, NY, USA) were used in order to view the images and to place circular regions of interest (ROIs) over the vertebral bodies from L1 to L5 in the sagittal plane, on T1-weighted images and on images obtained automatically on ADC maps (b0-b300, b0-b500, and b0-b800). The ROIs were drawn in the mid-anterior portion of each vertebral body, excluding the posterior vascular pole, cortical bone, intervertebral spaces, and vertebral spaces. Vertebral bodies affected by confounding signal alterations were excluded from the measurement. The means of the T1-weighted TSE intensity values and of the ADC values were then calculated for each patient. The mean values for the T1-weighted signal and the ADC (× 10^-3^ mm^2^/s), obtained from the measurements for each vertebral body, were used for the correlation with the BMD T-score. In addition to evaluating the sample as a whole, we also performed an analysis in which we compared the patients ≤ 60 years of age with those > 60 years of age.

### Statistical analysis

All statistical analyses were performed with the IBM SPSS Statistics software package, version 20.0 (IBM Corp., Armonk, NY, USA) or GraphPad Prism software, version 7.01 (GraphPad Software Inc., San Diego, CA, USA). Means of the ADC (b300, b500, and b800) and T1-weighted signal intensity values were obtained both for the subgroups of patients with normal BMD, osteoporosis, osteopenia and for the sample as a whole after stratification by age group. We used Pearson’s correlation coefficient (r) to evaluate correlations among the variables T-score, BMD, ADC (b300, b500, and b800), and T1-weighted signal intensity values, for the sample as a whole and for the two age groups. Values of *p* < 0.05 on two-tailed tests were considered significant.

We used one way analysis of variance to evaluate the correlations of the ADC with T1-weighted signal intensity values over the spectrum from normal BMD to osteopenia to osteoporosis. We used unpaired t-tests to evaluate the correlation between ADC and T1-weighted signal intensity for the two age groups.

## RESULTS

A total of 25 volunteers were recruited. Of those, 13 met the inclusion criteria. The mean age of the patients in our sample was 61 years (range, 56-72 years). The mean interval between DEXA and MRI was 11.2 days (range, 4-27 days). Images of the five vertebrae of interest were acquired and post-processed in almost all 13 cases, although one L5 vertebral body was excluded from the analysis because it was found to harbor a hemangioma (with no aggressive or suspicious characteristics). Therefore, 64 lumbar vertebrae were examined. The review of the examinations did not reveal any additional significant findings: in one case, facet syndrome was diagnosed but did not lead to exclusion of the patient. In the spinal tracts examined, there was no evidence of previous acute or subacute vertebral compression fractures or of previously unknown oncological diseases.

**Figure 1:**
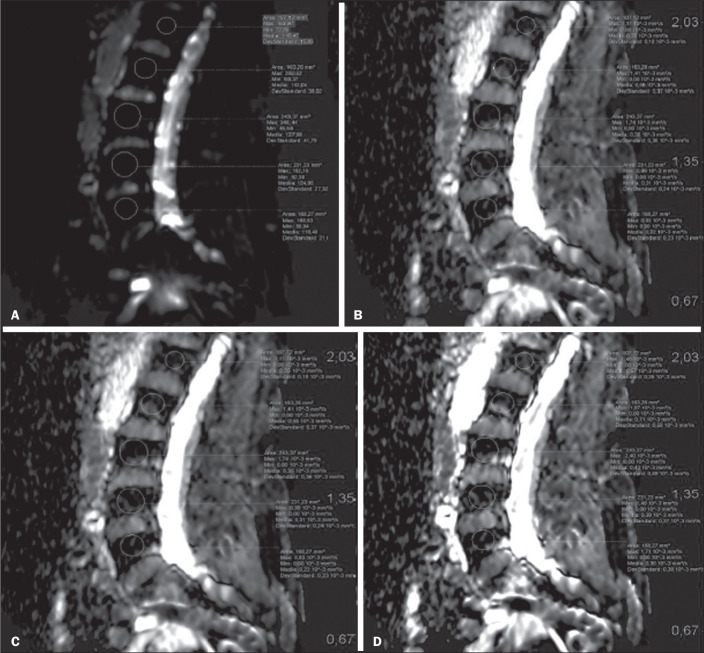
Examples of ROI placement on ADC maps: b0 (A); b300 (B); b500 (C); b800 (D).

We placed 226 circular ROIs. However, in two cases, the MRI was discontinued early because of patient claustrophobia. Therefore, for the 11 patients (84.6%) who underwent MRI with a complete protocol, including DWI sequences, a total of 162 ROIs were on the ADC maps generated by the software (Figure 1). On the T1-weighted images, which were available for all 13 patients, we placed a total of 64 ROIs to measure the vertebral body signal intensity.

On the basis of the DEXA data, four patients (30.8%) were diagnosed with osteoporosis (T-score < -2.5), whereas five patients (38.4%) had a T-score between -2.5 and -1 (osteopenia) and four (30.8%) had a T-score within the normal range (> -1). The data obtained are listed by BMD T-score, in descending order, in Table 2 and by patient age, in ascending order, in Table 3.

**Table 2:** Data for the sample as a whole, listed in descending order by T-score.^*^

Patient	Age (years)	T-score	BMD	ADC			T1
				b300	b500	b800	
3	56	-0.2	1.159	0.594	0.412	0.326	370.1
5	59	-0.5	1.12	0.6975	0.505	0.3725	322.0475
7	59	-0.7	1.098	0.752	0.548	0.454	359.08
2	53	-0.7	1.099	N/A	N/A	N/A	274.834
6	59	-1.6	0.985	0.784	0.594	0.446	289.524
12^†^	70	-1.6	N/A	0.786	0.758	0.428	455.4475
9	63	-1.9	0.947	0.672	0.482	0.338	302.4
8	59	-2.0	0.945	0.598	0.47	0.358	418.498
4	58	-2.2	0.914	0.766	0.592	0.46	385.234
10	65	-2.6	0.864	0.512	0.42	0.37	442.136
1	50	-2.8	0.846	1.258	0.828	0.574	541.348
11	66	-3.5	0.757	N/A	N/A	N/A	856.096
13	72	-3.8	0.745	0.74	0.48	0.356	419.656

* No shading = normal BMD; light gray shading = osteopenia; dark gray shading = osteoporosis.

† For patient no. 12, the femur BMD value was 0.884 g/cm^2^.

T1, T1-weighted signal intensity; N/A, not available.

**Table 3:** Data for the sample as a whole, listed in ascending order by patient age.^*^

Patient	Age (years)	T-score	BMD	ADC			T1
				b300	b500	b800	
1	50	-2,8	0,846	1,258	0,828	0,574	541,348
2	53	-0.7	1.099	N/A	N/A	N/A	274.834
3	56	-0.2	1.159	0.594	0.412	0.326	370.1
4	58	-2.2	0.914	0.766	0.592	0.46	385.234
5	59	-0.5	1.12	0.6975	0.505	0.3725	322.0475
6	59	-1.6	0.985	0.784	0.594	0.446	289.524
7	59	-0.7	1.098	0.752	0.548	0.454	359.08
8	59	-2	0.945	0.598	0.47	0.358	418.498
9	63	-1.9	0.947	0.672	0.482	0.338	302.4
10	65	-2.6	0.864	0.512	0.42	0.37	442.136
11	66	-3.5	0.757	N/A	N/A	N/A	856.096
12^†^	70	-1.6	N/A	0.786	0.758	0.428	455.4475
13	72	-3.8	0.745	0.74	0.48	0.356	419.656

* No shading = normal BMD; light gray shading = osteopenia; dark gray shading = osteoporosis.

† For patient no. 12, the femur BMD value was 0.884 g/cm^2^.

T1, T1-weighted signal intensity; N/A, not available.

The mean ADC values in the patients with osteoporosis were 0.83 × 10^-3^ (b0-b300), 0.57 × 10^-3^ (b0-b500), and 0.43 × 10^-3^ (b0-b800). The mean ADC values in the patients with osteopenia were 0.72 × 10^-3^ (b0-b300), 0.57 × 10^-3^ (b0-b500), and 0.40 × 10^-3^ (b0-b800). The mean ADC values in the patients with normal T-scores were 0.68 × 10^-3^ (b0-b300), 0.48 × 10^-3^ (b0-b500), and 0.38 × 10^-3^ (b0-b800). The mean T1-weighted signal intensity was 564.8 in the patients with osteoporosis, 370.2 in the patients with osteopenia, and 331.5 in the patients with normal T-scores. Those results are summarized in Table 4.

**Table 4:** ADC values and T1-weighted signal intensity among the 11 patients for whom those data were available.

Osteoporosis (n = 3)	Osteopenia (n = 5)	Normal BMD (n = 3)
b300		
0.512	0.784	0.594
1.258	0.786	0.6975
0.74	0.672	0.752
0.598		
0.766		
Mean, 0.836666667	Mean, 0.7212	Mean, 0.681166667
b500		
0.42	0.594	0.412
0.828	0.758	0.505
0.48	0.482	0.548
0.47		
0.592		
Mean, 0.576	Mean, 0.5792	Mean, 0.488333333
b800		
0.37	0.446	0.326
0.574	0.428	0.3725
0.356	0.338	0.454
0.358		
0.46		
Mean, 0.433333333	Mean, 0.406	Mean, 0.384166667
T1		
442.136	289.524	370.1
541.348	455.4475	322.0475
856.096	302.4	359.08
419.656	418.498	274.834
385.234		
Mean, 564.809	Mean, 370.2207	Mean, 331.515375

T1, T1-weighted signal intensity.

**Figure 2:**
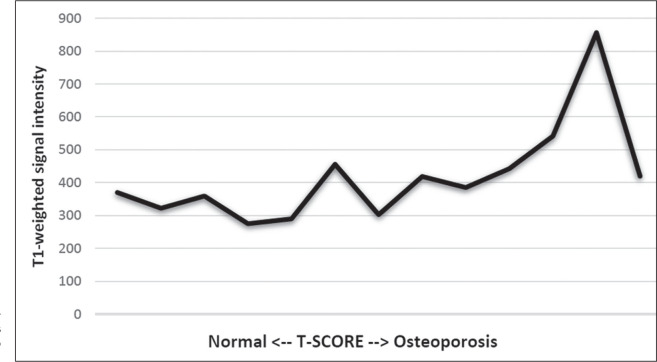
T1-weighted signal intensity over the spectrum of T-scores (from normal BMD to osteopenia to osteoporosis).

**Figure 3:**
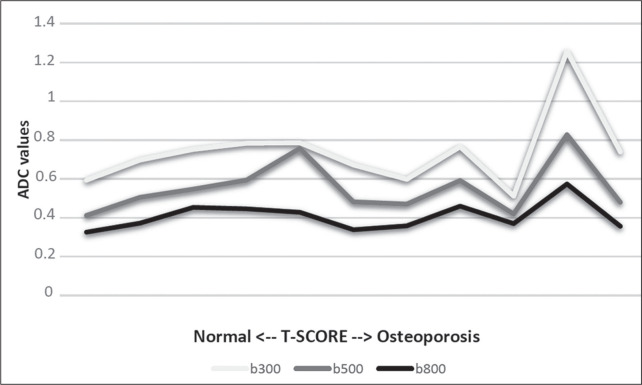
ADC values over the spectrum of T-scores (from normal BMD to osteopenia to osteoporosis).

When we divided the sample by patient age, there were eight patients in the ≤ 60-year group and five patients in the > 60-year group. As can be seen in Table 5, the mean ADC values in the ≤ 60-year group were 0.77 × 10^-3^ (b0-b300), 0.56 × 10^-3^ (b0-b500), and 0.42 × 10^-3^ (b0-b800), with a mean T1-weighted signal intensity value of 370.08, compared with 0.67 × 10^-3^ (b0-b300), 0.53 × 10^-3^ (b0-b500), and 0.37 × 10^-3^ (b0-b800), with a mean T1-weighted signal intensity value of 414.75, in the > 60-year group. The correlations among the variables are summarized in Table 6 for the sample as a whole and in Table 7 for the two age groups. In the sample as a whole, there was a significant inverse correlation between the mean T-score and the mean T1-weighted signal intensity (r = -0.634, *p* < 0.05; Figure 2), as well as inverse, less than significant, correlations between the T-score and the ADC values (Figure 3). In the ≤ 60-year and > 60-year groups, the mean T-score also correlated negatively with the mean T1-weighted signal intensity, although the correlations were weak (r = -0.688, *p* < 0.59 and r = -0.504, *p* < 0.387, respectively).

**Table 5:** Data for the sample as a whole, stratified by age group.^*^

Patient		Age (years)	T-score	BMD	ADC			T1
					b300	b500	b800	
≤ 60 years of age	1	50	-2.8	0.846	1.258	0.828	0.574	541.35
	2	53	-0.7	1.099	N/A	N/A	N/A	274.83
	3	56	-0.2	1.159	0.594	0.412	0.326	370.1
	4	58	-2.2	0.914	0.766	0.592	0.46	385.23
	5	59	-0.5	1.12	0.6975	0.505	0.3725	322.05
	6	59	-1.6	0.985	0.784	0.594	0.446	289.52
	7	59	-0.7	1.098	0.752	0.548	0.454	359.08
	8	59	-2.0	0.945	0.598	0.47	0.358	418.5
Mean		-	-	-	0.7785	0.564143	0.427214286	370.08
> 60 years of age	9	63	-1.9	0.947	0.672	0.482	0.338	302.4
	10	65	-2.6	0.864	0.512	0.42	0.37	442.14
	11	66	-3.5	0.757	N/A	N/A	N/A	856.1
	12	70	-1.6	N/A	0.786	0.758	0.428	455.45
	13	72	-3.8	0.745	0.74	0.48	0.356	419.66
Mean		-	-	-	0.6775	0.535	0.373	414.75

* No shading = normal BMD; light gray shading = osteopenia; dark gray shading = osteoporosis.

T1, T1-weighted signal intensity; N/A, not available.

**Table 6:** Correlations in the sample as a whole.

Variable		Statistic	T-score	BMD	ADC			T1
					b300	b500	b800	
T-score		r	1	0.999	-0.276	-0.182	-0.192	-0.634
		*p*	-	0.000	0.412	0.593	0.571	0.020
		n	13	12	11	11	11	13
BMD		r	0.999	1	-0.285	-0.261	-0.214	-0.653
		*p*	0.000	-	0.426	0.466	0.553	0.021
		n	12	12	10	10	10	12
ADC	b300	r	-0.276	-0.285	1	0.870	0.879	0.503
		*p*	0.412	0.426	-	0.000	0.000	0.115
		n	11	10	11	11	11	11
	b500	r	-0.182	-0.261	0.870	1	0.862	0.521
		*p*	0.593	0.466	0.000	-	0.001	0.100
		n	11	10	11	11	11	11
	b800	r	-0.192	-0.214	0.879	0.862	1	0.480
		*p*	0.571	0.553	0.000	0.001	-	0.135
		n	11	10	11	11	11	11
T1		r	-0.634	-0.653	0.503	0.521	0.480	1
		*p*	0.020	0.021	0.115	0.100	0.135	-
		n	13	12	11	11	11	13

T1, T1-weighted signal intensity.

**Table 7:** Correlations in the sample, by age group.

Age group	Variable		Statistic	T-score	BMD	ADC			T1
						b300	b500	b800	
≤ 60 years of age (n = 8)	T-score		r	1	1.000	-0.657	-0.751	-0.707	-0.688
			*p*	-	0.000	0.109	0.052	0.076	0.059
			n	8	8	7	7	7	8
	BMD		r	1.000	1	-0.658	-0.755	-0.713	-0.678
			*p*	0.000	-	0.108	0.050	0.072	0.065
			n	8	8	7	7	7	8
	ADC	b300	r	-0.657	-0.658	1	0.979	0.932	0.702
			*p*	0.109	0.108	-	0.000	0.002	0.079
			n	7	7	7	7	7	7
		b500	r	-0.751	-0.755	0.979	1	0.971	0.640
			*p*	0.052	0.050	0.000	-	0.000	0.121
			n	7	7	7	7	7	7
		b800	r	-0.707	-0.713	0.932	0.971	1	0.569
			*p*	0.076	0.072	0.002	0.000	-	0.183
			n	7	7	7	7	7	7
	T1		r	-0.688	-0.678	0.702	0.640	0.569	1
			*p*	0.059	0.065	0.079	0.121	0.183	-
			n	8	8	7	7	7	8
> 60 years of age (n = 5)	T-score		r	1	0.996	0.084	0.567	0.446	-0.504
			*p*	-	0.004	0.916	0.433	0.554	0.387
			n	5	4	4	4	4	5
	BMD		r	0.996	1	-0.387	-0.074	-0.473	-0.638
			*p*	0.004	-	0.747	0.953	0.686	0.362
			n	4	4	3	3	3	4
	ADC	b300	r	0.084	-0.387	1	0.736	0.400	0.032
			*p*	0.916	0.747	-	0.264	0.600	0.968
			n	4	3	4	4	4	4
		b500	r	0.567	-0.074	0.736	1	0.871	0.366
			*p*	0.433	0.953	0.264	-	0.129	0.634
			n	4	3	4	4	4	4
		b800	r	0.446	-0.473	0.400	0.871	1	0.735
			*p*	0.554	0.686	0.600	0.129	-	0.265
			n	4	3	4	4	4	4
	T1		r	-0.504	-0.638	0.032	0.366	0.735	1
			*p*	0.387	0.362	0.968	0.634	0.265	-
			n	5	4	4	4	4	5

T1, T1-weighted signal intensity.

## DISCUSSION

Osteoporosis is the most common cause of vertebral compression fracture^(1)^. Even when such fractures are treated aggressively, morbidity and mortality remain high^(2)^, with reduced life expectancy, impaired quality of life^(3-5)^, and estimated costs of approximately €75 billion projected for the year 2050 in Europe^(3,6)^.

The complex pathophysiological modifications underlying the increased bone fragility due to osteoporosis are not yet well understood, especially because of the diagnostic limitations to conducting *in vivo* studies of this pathology^(2,7)^. In the United States, an estimated 44 million people suffer from osteoporosis, which affects approximately 55% of the population over 50 years of age^(2,7)^.

Osteoporosis has been defined as “a disease characterized by low bone mass and microarchitectural deterioration of bone tissue, leading to enhanced bone fragility and a consequent increase in fracture risk”^(8)^. DEXA is used as a noninvasive technique for the quantification of osteoporosis prior to therapeutic interventions, given that BMD correlates with bone strength and is still the single best predictor of fracture risk^(3,9,10)^, thus being the reference parameter for the screening and follow-up of osteoporosis. The risk of vertebral fracture has been shown to increase by 60% for each standard deviation reduction in BMD^(11,12)^. Because DEXA allows the determination of only a single parameter (BMD), it leaves a large “gray area” regarding many qualitative aspects of the pathogenesis of osteoporosis^(13,14)^.

Despite the existence of a direct correlation between the BMD T-score and fracture risk, it is estimated that half of postmenopausal women who suffer a fracture caused by low-energy trauma have a BMD that is above the World Health Organization threshold for the diagnosis of osteoporosis, which is defined as a T-score equal to or greater than -2.5^(1,15)^. The BMD alone does not reflect bone quality, which also significantly affects bone strength and fracture risk^(3,15-17)^. Bone strength is in fact determined by qualitative factors such as trabecular architecture, progressive accumulation of bone damage (e.g., microfractures), bone thickness, the geometry of the cortical bone, osteon turnover, osteocyte density, cell viability, and composition of the bone marrow^(3,18,19)^.

In the present study, we focused on the role of MRI in bone marrow imaging. Some authors have highlighted a correlation between BMD and fat content in the bone marrow, proposing that the latter is a specific indicator of reduced bone strength^(20)^ and suggesting that it plays a crucial role in the physiopathology of osteoporosis. The increase in vertebral fat with aging has been demonstrated in various quantitative histological studies of vertebral biopsies and has been shown to be more pronounced in patients with osteoporosis^(11,21,22)^.

High-field MRI scanners can be particularly beneficial in DWI^(23)^. A 3.0-T scanner can provide images with a higher signal-to-noise ratio, resulting in better spatial, temporal, and spectral resolution. A higher signal-to-noise ratio also provides greater sensitivity for minimal variations in the ADC, thus increasing the accuracy of the measurements on the ADC maps^(24)^.

There is evidence that the mean ADC values for the lumbar spine are significantly lower in patients with osteoporosis than in those with normal BMD. Through the use of DWI in 3.0-T scanners, He et al.^(25)^ found a significant positive correlation between ADC values and BMD, as did Momeni et al.^(26)^, who also proposed an ADC cutoff value of 400 (× 10^-6^ mm^2^/s) for the diagnosis of osteoporosis.

In a prospective study employing MR spectroscopy and DWI sequences in postmenopausal women, Agrawal et al.^(27)^ concluded that DWI sequences are useful for evaluating changes in bone marrow content. The authors identified significant increases in ADC values with the increase in vertebral fat content in patients with osteoporosis, as well as a significant positive correlation between T-scores and ADC, together with a negative correlation between adipose bone marrow fat content and BMD/ADC values. Capuani et al.^(23)^ proposed the coexistence of two mechanisms of opposite significance involved in the structural alteration of osteoporotic bone. The first, attributed to the reduction in bone trabeculae, would consist in the increase of the interstitial spaces due to increases in the size of the pores and of the canaliculi, resulting in greater diffusion of water and an increase in the ADC values. The second, opposite, mechanism, due to the increase in yellow bone marrow, would be narrowing of the interstitial spaces with restricted diffusion and lower ADC values reduction: the prevalence of one or the other would determine the ADC value obtained through DWI.

Yeung et al.^(14)^ and Tang et al.^(15)^ both observed that a decrease in BMD and an increase in bone marrow fat content correspond to a proportional reduction in the ADC value in postmenopausal patients. It was therefore hypothesized that when yellow bone marrow fills the free spaces left by trabeculae deterioration and a loss of red marrow, there is a proportional decrease in the ADC values due to greater extracellular water restriction (compression of water by hydrophobic yellow bone marrow in the intertrabecular spaces), together with an overall reduction in the aqueous content, which is proportionally lower in the yellow marrow than in the red marrow^(2,15)^. Ward et al.^(28)^ and Nonomura et al.^(29)^ argued that the ADC value of red marrow is higher than is that of yellow marrow.

Further confirming what Yeung et al.^(14)^ suggested, Fanucci et al.^(11)^ found that ADC values among patients with osteoporosis were significantly lower in those who were postmenopausal than in those who were premenopausal. The authors argued that the restricted diffusion in the vertebral bodies of elderly individuals can be considered an earlier and more sensitive indicator of structural bone alteration than is the BMD T-score^(11,14)^. Hatipoglu et al.^(2)^ demonstrated a direct correlation between T-scores and ADC values. However, they reported a stronger correlation between the T-score and the T1-weighted signal intensity, suggesting that the latter plays a significant role as an early indicator of structural alteration in osteoporotic bone and once again emphasizing the central role of yellow bone marrow in the pathogenesis of osteoporosis. Nevertheless, there is no clear consensus in the literature. Koyama et al.^(10)^ reported observations similar to those of Hatipoglu et al.^(2)^ regarding the usefulness of T1-weighted imaging, showing an inverse correlation between ADC values and osteoporosis, a result consistent with ours. In contrast, Griffith et al.^(30)^ found no significant correlation between the ADC values and the diagnosis of osteoporosis. In the present study, we found a progressive increase in the ADC values over the spectrum from normal BMD to osteopenia to osteoporosis. However, the differences among the patients with osteoporosis, those with osteopenia, and those with normal BMD were not statistically significant. In our sample, the mean T1-weighted signal intensity was also higher among the patients with osteoporosis than among those with osteopenia, in turn being higher among the patients with osteopenia than among those with normal BMD; again, the differences among those groups were not statistically significant. However, when we stratified the sample by age, we observed that the ADC values were lower in the patients > 60 years of age, whereas T1-weighted signal intensity was lower in the patients ≤ 60 years of age.

Our results underscore the importance of the T1-weighted sequences in MRI of the bone marrow and are therefore in agreement with those of other authors^(31,32)^. In fact, scores based on T1-weighted MRI have been proposed to diagnose and characterize osteoporosis, including the M-score, devised by Bandirali et al.^(33)^, and the vertebral bone quality score, devised by Ehresman et al.^(34)^.

In the present study, the strongest correlation was the inverse correlation between the T-score and the T1-weighted signal intensity, the latter being higher in patients with osteoporosis than in those with osteopenia and those with normal BMD, as well as in being higher in the patients > 60 years of age. However, contrary to our working hypothesis, we observed a weak inverse correlation between the T-score and the ADC values. Nevertheless, a careful analysis of the correlations after the stratification of the sample by age revealed an interesting finding: a direct correlation between the T-score and the ADC values in the in the patients > 60 years of age. In addition, the mean ADC values were lower in those patients. These results are in agreement with our initial hypothesis.

The inverse correlation between the ADC values and T-scores in the patients ≤ 60 years of age (who predominated in our sample) could be explained by the first mechanism described by Capuani et al.^(23)^: in an early phase after menopause, a reduction in the size of the vertebral bone trabeculae may not correspond to an increase in yellow marrow sufficient to cause appreciable restricted diffusion, probably because bone marrow perfusion is more efficient in younger people.

Multiple factors, some of which might be unknown, could affect the diffusion coefficient measured on spinal MRI: vertebral perfusion, as suggested by Koyama et al.^(10)^, is a determinant of the ADC value which is difficult to assess and the bone marrow fat content can vary according to the sex of the subject and individual constitutional factors^(30,35)^.

### Limitations

Our study has some limitations. Primarily, the small sample size limited the statistical power of the study. In addition, we selected only postmenopausal patients, whereas a comparison with premenopausal subjects could have been useful. Furthermore, we classified the patients as having osteoporosis, osteopenia, or normal BMD solely on the basis of the T-score. That could be a source of error, given that, as previously stated, BMD, with its intrinsic limitations, may not reflect the real degree of bone alteration. The measurements made by MRI could in fact have reflected tissue alterations that are totally or partially unrelated to the BMD. Moreover, we did not exclude patients already under treatment for osteoporosis, which could represent a confounding factor.

## CONCLUSIONS

We believe that it will not be possible to replace DEXA in the diagnostic workup of osteoporosis in the near future. However, our results and data in the literature demonstrate that MRI could provide additional information that could improve understanding of the qualitative mechanisms underlying the disease. Such information would complement the quantitative data obtainable through the determination of BMD alone^(3,18)^.

Although DWI sequences are useful for studying the pathophysiology of osteoporosis, T1-weighted sequences are more sensitive for the identification of structural alterations in vertebral bodies. We believe that measuring the signal intensity of vertebral bodies on T1-weighted images could be beneficial in the screening for and diagnostic workup of osteoporosis.
